# Cure of a CD20-positive peripheral T-cell lymphoma-NOS with CHOPE regimen plus surgery a case report and literature review

**DOI:** 10.3389/fmed.2025.1669823

**Published:** 2025-11-26

**Authors:** Chenyu Chi, Shuo Wang, Man Xu, Yue Shi, Xuehong Ran

**Affiliations:** Weifang People’s Hospital, Shandong Second Medical University, Weifang, Shandong, China

**Keywords:** peripheral T-cell lymphoma not otherwise specified, aberrant CD20 expression, TCR gene rearrangement, CHOPE regimen, rituximab

## Abstract

Peripheral T-cell lymphoma non-specific type (PTCL-NOS) is the most common subtype of mature T-cell tumors, which is clinically characterized by high invasiveness and poor prognosis. It is worth noting that PTCL-NOS rarely abnormally expresses B-cell antigens (such as CD20). This rare immunophenotype makes it easily confused with B-cell non-Hodgkin’s lymphoma (B-NHL), which brings challenges to clinical diagnosis and may lead to wrong treatment decisions. At present, CHOP regimen, as the first-line standard therapy for PTCL-NOS, has the limitations of low complete remission rate and high recurrence risk. In order to improve the efficacy, recent studies have confirmed that the combination of etoposide (CHOPE regimen) on the basis of CHOP regimen can significantly improve the treatment response for newly diagnosed patients aged ≤ 65 years. This paper reports a rare case of PTCL-NOS with abnormal expression of CD20 in the tongue. The patient received four cycles of CHOPE regimen after surgical resection of the primary tumor and underwent PET/CT mid-term evaluation. Through this case, we aim to further explore the pathological diagnosis difficulties of such rare cases and preliminarily evaluate the clinical application value of CHOPE regimen.

## 1 Introduction

Peripheral T-cell lymphoma (PTCL) represents a group of highly heterogeneous and aggressive lymphoid malignancies with a generally poor prognosis. According to the World Health Organization (WHO) classification, PTCL can be categorized into disseminated/leukemic, nodal, extranodal, and cutaneous subtypes. PTCL accounts for approximately 10%–15% of all non-Hodgkin lymphomas (NHL). The major subtypes, in descending order of incidence, are PTCL, not otherwise specified (PTCL-NOS, 25.9%), angioimmunoblastic T-cell lymphoma (AITL, 18.5%), extranodal NK/T-cell lymphoma, nasal type (ENKTL, 10.4%), and adult T-cell leukemia/lymphoma (ATLL, 9.6%), followed by anaplastic large cell lymphoma (ALCL, ALK-positive and ALK-negative combined, 12.1%) (1). Although PTCL-NOS is the most common subtype, it lacks specific pathological features. It typically presents with a T-cell immunophenotype, and only in extremely rare cases does it aberrantly express B-cell antigens such as CD20. Given the significant differences in prognosis and treatment strategies between T-cell and B-cell lymphomas, accurately distinguishing CD20-positive PTCL from B-cell lymphoma is crucial. This distinction relies on a comprehensive interpretation of morphological, immunohistochemical, and molecular findings.

Gene rearrangement analysis is an effective ancillary tool for the diagnosis of lymphoma, particularly for T-cell lymphoma (TCL) that lack a specific immunophenotype. The molecular structure of the T-cell receptor (TCR) is encoded by various gene segments, including variable (V), diversity (D), joining (J), and constant (C) regions. During the maturation of lymphocytes, these TCR genes undergo a somatic recombination process known as V(D)J rearrangement. This process begins with the recombination of a D and a J gene segment, followed by the joining of a V segment to the rearranged DJ complex. This ultimately forms the complementarity-determining region 3 (CDR3), which is critical for antigen recognition. Furthermore, the random insertion and deletion of nucleotides at the V-D and D-J junctions, a process called N-nucleotide addition, significantly enhances the diversity of the CDR3 sequence (2). Since malignant neoplasms are generally of monoclonal origin, all tumor cells within a single patient share an identical TCR gene rearrangement sequence. Consequently, when a definitive diagnosis cannot be established by histopathology alone, the results of gene rearrangement analysis become particularly crucial.

Herein, we report the case of a middle-aged female who presented with a lingual mass. Intraoperative pathology and immunohistochemistry (IHC) revealed an immunophenotype consistent with both T-cell and B-cell lymphoma. With the aid of gene rearrangement analysis, a definitive diagnosis of PTCL-NOS with aberrant CD20 expression was established. The patient subsequently achieved a complete response (CR) as assessed by an interim PET/CT scan following four cycles of CHOPE chemotherapy.

## 2 Case presentation

### 2.1 Patient condition

A 62-year-old female was admitted to our hospital with a chief complaint of a “mass on the right side of the tongue for over 3 months.” The patient had incidentally discovered the mass on her right lingual margin, which progressively enlarged and was accompanied by restricted tongue mobility, prompting her to seek medical attention.

A physical examination revealed a firm, well-demarcated mass measuring approximately 4.0 cm × 3.0 cm on the right ventral surface of the tongue, with poor mobility. The patient had a past medical history of gastroesophageal reflux disease but no family history of cancer and denied B symptoms (fever, night sweats, or weight loss). Initial laboratory tests indicated mild anemia (Hemoglobin: 108 g/L) and elevated liver enzymes (ALT: 99 U/L, AST: 66 U/L).

### 2.2 Diagnosis and investigations

**FIGURE 1:**
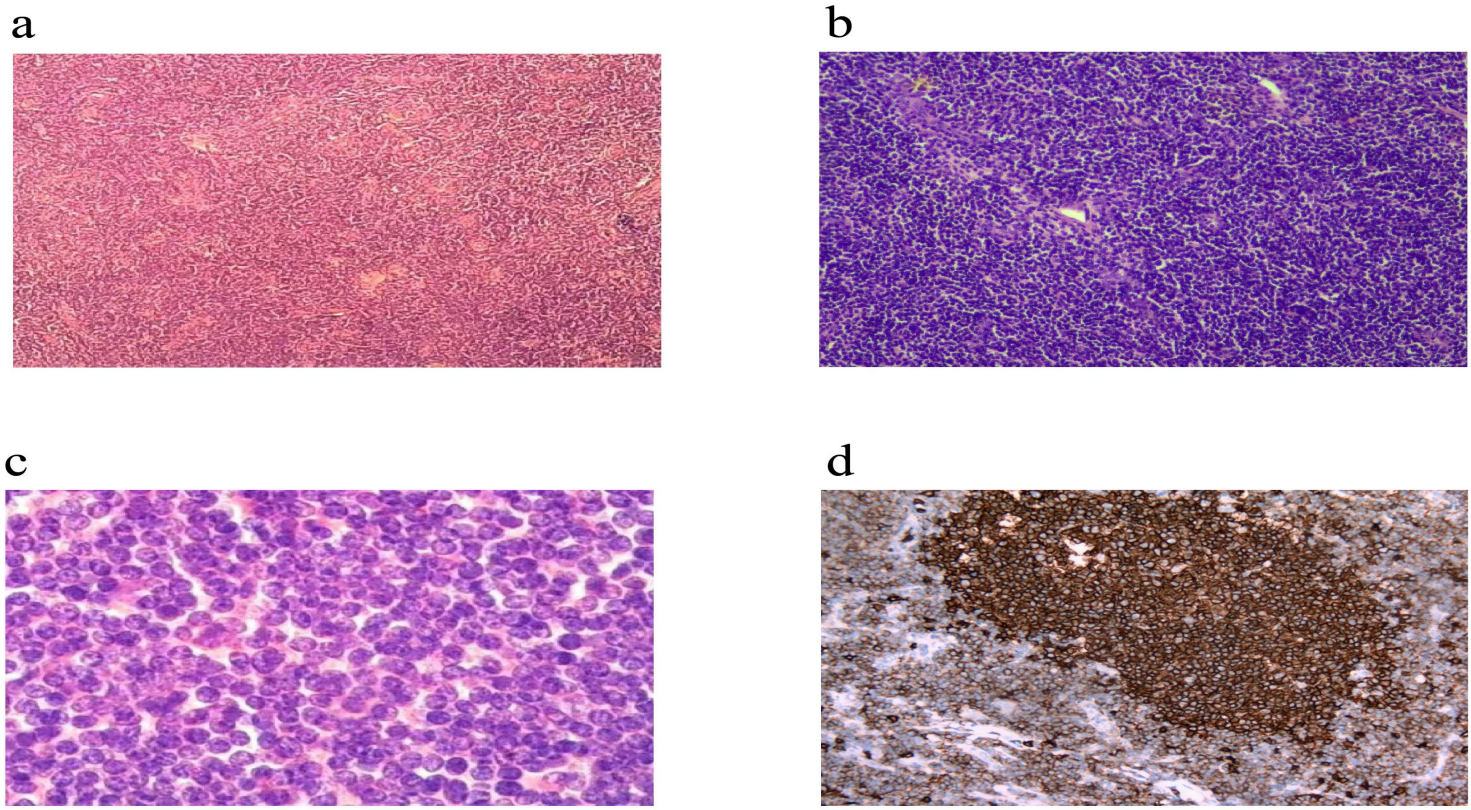
Histopathological features of the tongue root mass biopsy (H&E stain). The micrographs show that the tumor tissue exhibits a diffuse, sheet-like growth pattern. The tumor tissue has involved a lingual tonsil and destroyed its architecture. **(a)** Low-power view (H&E, × 100): A dense infiltration of tumor cells is observed, with only a few scattered residual lymphoid follicles remaining. **(b)** Medium-power view (H&E, × 200): The tumor consists of a relatively monotonous population of medium-sized, atypical lymphoid cells, with a background of scattered non-neoplastic inflammatory cells. **(c)** High-power view (H&E, × 400): The cytological features of the tumor cells are clearly demonstrated. The cells have irregular, convoluted, or cerebriform nuclear contours, coarse and hyperchromatic chromatin, inconspicuous nucleoli, and scant cytoplasm. **(d)** Immunohistochemistry (IHC) for CD20 (IHC, × 200): The neoplastic cells exhibit diffuse, uniform, and strong positive expression. The brown chromogen is clearly localized to the cell membrane, circumferentially outlining the blue, hematoxylin-counterstained nuclei.

The patient underwent a partial glossectomy. Intraoperative frozen section pathology suggested a lymphoproliferative disorder, favoring a hematopoietic or lymphoid malignancy (Figure 1).

Postoperative paraffin-embedded sections and IHC revealed that tumor cells were extensively positive for T-cell markers **CD3, CD2, CD5, and CD43**, and concurrently showed strong positivity for the B-cell marker **CD20**. Other markers included BCL-2 (+, 85%), with sparse or scattered positivity for CD79α, CD10, PAX-5, and CD19. The Ki-67 proliferation index was approximately 30%. *In situ* hybridization for EBER was negative.

To resolve the diagnostic ambiguity, gene rearrangement studies were performed. TCR gene analysis demonstrated a monoclonal rearrangement in the TCRB gene, while the B-cell receptor (BCR) genes (IGH/IGK) were polyclonal.

Following a consultation with a referral center, the diagnosis was confirmed as PTCL-NOS with aberrant CD20 expression. Supplementary IHC staining was negative for CD34, TdT, CD7, CD30, and Perforin.

### 2.3 Staging and treatment

**FIGURE 2:**
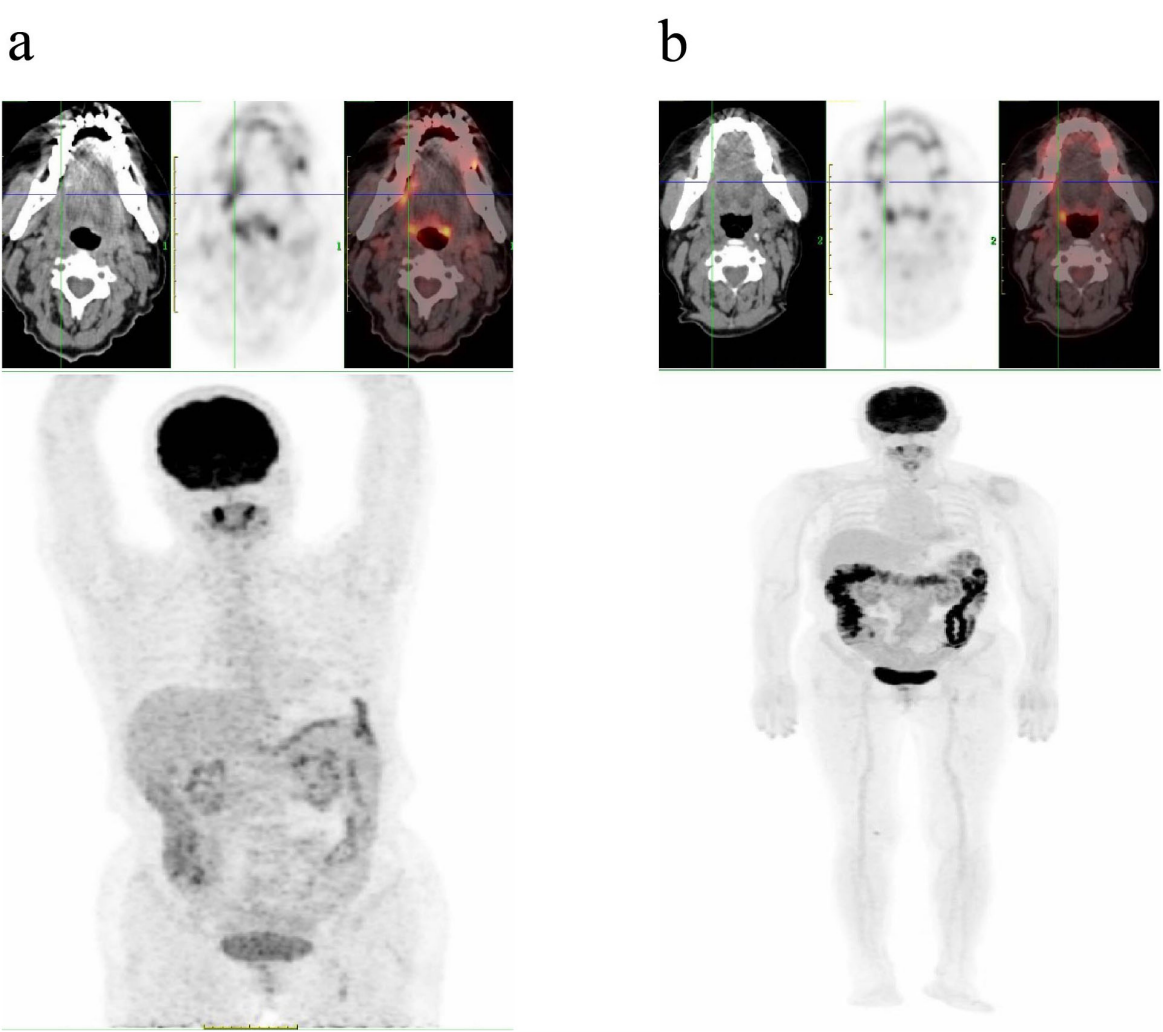
**(a)** An initial PET/CT scan (4 October 2024) identified a hypermetabolic lesion on the right lateral border of the tongue. **(b)** A follow-up PET/CT scan (5 March 2025) demonstrated a complete metabolic response, with no evidence of abnormal FDG uptake at the previously involved site.

For systemic staging, a PET/CT scan was conducted, which reported: (1). Markedly increased metabolic activity in the thickened anterior and bilateral walls of the oropharynx (SUVmax = 15.8); (2). Scattered lymph nodes in the bilateral neck, some with slightly elevated metabolic activity (SUVmax = 4. 3); (3). No definitive tumor-related hypermetabolism was observed in the postsurgical site of the tongue or elsewhere (Figure 2a).

A bone marrow biopsy and aspirate were negative for lymphoma involvement. The final diagnosis was PTCL-NOS, Ann Arbor Stage I, with an International Prognostic Index (IPI) score of 1 (Low Risk).

The patient was initiated on the CHOPE chemotherapy regimen (Cyclophosphamide 1,100 mg on day 1, Doxorubicin 60 mg on day 1, Vincristine 2 mg on day 1, Etoposide 100 mg on days 1–3, and Prednisone 50 mg twice daily on days 1–5), along with supportive care.

An interim PET/CT scan (Figure 2b) performed after four cycles of CHOPE showed a complete resolution of the hypermetabolic lesions previously seen in the oropharynx and neck. The scan revealed no abnormal metabolic activity throughout the body, consistent with a CR and a Deauville score of 1. The patient demonstrated a significant response to treatment and is currently continuing the planned therapeutic course.

## 3 Literature review and analysis

### 3.1 Methods

A systematic literature review was conducted to identify all published cases of CD20-positive PTCL. We searched the PubMed, Web of Science, Embase, and China National Knowledge Infrastructure (CNKI) databases for articles published from their inception to April 2024. The search strategy combined terms for T-cell lymphoma (“Peripheral T-cell Lymphoma” OR “T-cell Lymphoma” OR “TCL” OR “PTCL”) with the term “CD20.” No language restrictions were applied. Additionally, the reference lists of relevant review articles were manually screened to identify further eligible studies. Inclusion criteria were as follows: (1) a histopathologically confirmed diagnosis of PTCL; (2) definitive data on CD20 expression by immunohistochemistry; and (3) availability of individual patient’s clinical information, including treatment and outcome. Exclusion criteria were: (1) duplicate publications; (2) review articles, editorials, expert opinions, or letters; and (3) studies from which individual case data could not be extracted. Data on patient demographics, PTCL subtype, treatment regimens, therapeutic response, and clinical outcomes were extracted from the included articles.

### 3.2 Results

Following the screening of titles, abstracts, and full texts, a total of 20 articles met our inclusion criteria, encompassing 28 patients with CD20-positive PTCL. The clinical characteristics, treatment details, and outcomes of these patients, along with the present case, are summarized in Table 1.

This study involved a retrospective analysis of the clinical data from 29 patients with CD20 + PTCL-NOS. The baseline characteristics of the patients were as follows: 18 males and 11 females, with a male-to-female ratio of approximately 2:1. The median age at diagnosis was 66.5 years, indicating a predilection for middle-aged and elderly males. Lymphadenopathy was the most common clinical presentation, with the disease primarily affecting lymph nodes (nodal involvement). However, a significant proportion of patients also presented with extranodal involvement. The affected sites were diverse, including the gastrointestinal tract, parotid gland, base of the tongue, skin, and uterine cervix. The overall prognosis for this cohort of CD20 + PTCL-NOS patients was exceptionally poor. Among the evaluable cases, only 17.2% (5/29) of patients achieved PR or better. During the follow-up period, the disease-related mortality rate was 51.7% (15/29). The final outcomes for another31.0% (9/29) of patients could not be determined due to a short follow-up duration.

To evaluate the impact of first-line therapies on prognosis, patients were stratified based on the treatment regimen received: CHOP, R-CHOP, or CHOPE. Seven cases were excluded from this sub-analysis due to unknown or non-standard treatment protocols (Cases 2, 5, 8, 19, 20, 22, and 24). One patient (Case 10), who initially received CHOP but was predominantly treated with the CHOPE regimen, was classified within the CHOPE cohort for this analysis. Patients treated with the CHOP regimen (*n* = 9, median age 64 years) exhibited the poorest outcomes, with a mortality rate of 66.7% (6/9). Notably, two patients in this group (Cases 4 and 7) achieved disease control or remission following the addition of rituximab to a subsequent salvage therapy, suggesting a potential efficacy for rituximab in chemotherapy-refractory settings. In contrast, patients receiving first-line R-CHOP (*n* = 6, median age 66.5 years) demonstrated a markedly improved prognosis. The mortality rate in this cohort was substantially lower at 33.3% (2/6), while the objective response rate (ORR) reached 50.0% (3/6). The prognosis for patients treated with the intensified CHOPE regimen (*n* = 7, median age 62 years) was intermediate between the other two groups, with a mortality rate of 57.1% (4/7).

## 4 Discussion

CD20 is a 35–37 kDa transmembrane protein that appears on the surface of B lymphocytes before the expression of immunoglobulin (23). It is widely expressed on various B-cell populations, including centroblasts, centrocytes, mantle cells, marginal zone cells, and most immunoblasts. In benign lymphoid tissue, CD20 is strongly expressed in the germinal centers, with expression gradually diminishing outside this area. A significant expansion of the CD20-positive zone, presenting in a diffuse or nodular pattern, is highly suggestive of B-NHL. Conversely, marked atrophy, compression, or disappearance of the CD20-positive area indicates a non-B-cell NHL lesion. Therefore, CD20 positivity serves as a first-line marker for the diagnosis of B-cell lymphomas.

The occurrence of CD20-positive PTCL-NOS is clinically rare. The pathogenesis of CD20 + TCL remains unclear, but three main hypotheses have been proposed (4, 5): (1). CD20+ T-cells may originate from a subset of circulating T-cells undergoing neoplastic transformation. (2). CD20 positivity might be a marker of T-cell activation or proliferation. (3). T-lymphocytes may acquire the CD20 antigen through phagocytosis of surrounding B-cells. Due to this aberrant expression, CD20+ TCL is often misdiagnosed as B-NHL. A definitive diagnosis typically relies on a comprehensive analysis combining immunophenotyping and gene rearrangement studies. CD20+ TCLs usually exhibit positivity for CD3 and a monoclonal TCR gene rearrangement, whereas they often lack CD5, CD7, CD19, CD79α, and monoclonal IgH gene rearrangements. The absence of CD5 and CD7 is considered a typical histopathological feature (24).

CD20+ PTCL-NOS is more commonly observed in elderly male patients. Its prominent clinical features include multi-regional lymphadenopathy, a good ECOG performance status (0–1), but advanced stage (III/IV) disease and a high International Prognostic Index (IPI) score (≥3), indicating a highly aggressive biological behavior (5). Since the 1970’s, the CHOP regimen has been the standard of care for PTCL, but it has not sufficiently improved long-term outcomes. A multicenter retrospective study further confirmed that CD20 positivity is an independent adverse prognostic factor for progression-free survival (PFS) in patients with PTCL-NOS, though it holds no significant predictive value for overall survival (OS) (25). Consequently, the aberrant expression of CD20 may have important therapeutic implications, suggesting that anti-CD20 monoclonal antibodies could be a potential treatment option.

In CD20+ B-cell lymphomas, anti-CD20 monoclonal antibodies eliminate B-cells through at least four distinct mechanisms (26): (1) direct cell death, (2) antibody-dependent cell-mediated cytotoxicity (ADCC), (3) phagocytosis, and (4) complement-dependent cytotoxicity (CDC). These functional differences help classify anti-CD20 antibodies into Type I or Type II. Rituximab, a classic Type I antibody, is characterized by high CDC activity and its ability to sequester CD20 into lipid rafts, leading to B-cell lysis (27). However, it remains uncertain whether similar mechanisms are at play in the treatment of CD20+ PTCL.

Recent studies have shown that for newly diagnosed patients aged ≤ 65 years, adding etoposide to the CHOP regimen (CHOPE) significantly improves event-free survival (28). Etoposide primarily functions by interfering with topoisomerase II, which induces DNA strand breaks, inhibits mitosis, and causes cell cycle arrest in the late S or early G2 phase (29).

We compared the clinical characteristics of 28 patients with pathologically confirmed CD20+ PTCL-NOS who had complete clinical data available (including primary site, treatment regimen, and follow-up results). These characteristics are detailed in Table 1. These results further confirm that CD20+ PTCL-NOS is a lymphoma subtype characterized by high aggressivity and a poor prognosis. Through a comparative analysis of different treatment protocols, we found that the addition of rituximab can significantly improve patient survival. However, even within the R-CHOP group, some patients experienced disease relapse or progression, indicating a heterogeneity in the efficacy of rituximab. We speculate that this may be closely related to biological factors, such as the intensity and homogeneity of CD20 antigen expression on the surface of tumor T-cells, which warrants further elucidation.

**TABLE 1:** Treatment and prognostic characteristics of 28 patients with CD20+ Peripheral T-cell lymphoma, not otherwise specified (PTCL-NOS).

Cage	Age/sex	Clinical presentation	Treatment	IHC	Follow-up
1 (3)	54/M	Generalized superficial lymphadenopathy	CHOP × 3	CD3(+), CD5(+), CD4(+), CD20(diffuse, strong+), Ki-67 index: 40%.	Died (organ failure)
2 (3)	48/M	Generalized superficial lymphadenopathy, splenomegaly, weight loss of 25 kg in 3 months	R-DHAO	CD3(+), CD4(+), CD20(diffuse, strong+),MUM-1(+), bcl-2(+), Ki-67 index: 70%.	Alive (8 months)
3 (4)	71/M	Cervical mass	Chemotherapy (anthracycline-based), radiotherapy	CD2(+), CD3(+), CD5(+), CD7(+), CD4(+), CD20(+), CD30(dim,subset, +), CD56(dim, +).	Died (66 months), developed EBV + BCL at 50 months
4 (4)	77/M	Retroperitoneal lymphadenopathy	Chemotherapy (anthracycline-based), rituximab, radiotherapy	CD2(+), CD3(+), CD5(+), CD4(+), CD20(dim, +).	Alive (4 months)
5 (4)	75/M	Cervical lymphadenopathy	Chemotherapy	CD3(+), CD8(+), CD20(subset, +), Perforin(+), Granzyme B(+), CD30(dim to bright, +), CD56(dim, +).	Died (6 months)
6 (4)	36/F	Generalized superficial lymphadenopathy	Chemotherapy (anthracycline-based), rituximab; HSCT at month 14	CD2(+), CD3(+), CD5(+), CD7(dim, +), CD4(+), CD20(dim, +).	Died(16 months), lung mets at 8 months
7 (4)	75/M	Generalized superficial lymphadenopathy, pulmonary nodules	Chemotherapy (anthracycline-based), rituximab	CD3(+), CD7(dim, +), CD20(+), CD19(+), CD79a(+), CD30(rare, +).	Remission (10 months)
8 (4)	37/F	Bilateral parotid gland enlargement	Extended parotidectomy, corticosteroids	CD2(+), CD3(+), CD5(+), CD20(dim, +), FMC7(dim, +), CD30(rare, +).	AWD (18 months)
9 (5)	69/M	Abdominal distension, generalized superficial lymphadenopathy, splenomegaly, bone marrow involvement	CHOPE × 2	CD2(+), CD3(+), CD5(+), CD20+, Ki-67 index: 10%.	Alive (23 months)
10 (5)	48/M	Left abdominal mass, generalized superficial lymphadenopathy, splenomegaly, bone marrow involvement	CHOP × 1, CHOPE × 3, BV + CHPE × 2, Azacitidine + Chidamlde, Azacitidine + Chidamlde + BTZ + CTX	CD2(+), CD3(+), CD5(+), CD7(dim, +), CD20 (mid, +), Bcl-6(slight, +), CD30(dim to mid, +), Ki-67 index:20%.	Alive (14 months)
11 (5)	61/F	Chest tightness, cough, left supraclavicular subcutaneous mass	CHOPE × 4, GemoxD × 2, radiotherapy, R-GemoxD × 4, Azacitidine + Chidamlde	CD2(+), CD3(+), CD4(slight, +), CD20(+), MUM1(slight dim, +), Bcl-6(+), Ki-67 index:35%.	Died (16 months), CNS progression at 14 months
12 (6)	62/F	Nasal discharge, generalized superficial lymphadenopathy, splenomegaly	CHOEP × 4	CD2(+), CD3(+), CD5(+), CD7(+), CD8(+), CD20(+), CD45(+), UCHL-1(+), Bcl-2(+).	Died (8 months)
13 (7)	44/M	Giant gastric antral ulcer	Initially diagnosed as DLBCL, R-CHOP × 4, ulcer enlarged; total gastrectomy and regional lymph node dissection; CHASE × 3	CD3(+), CD4(+), CD5(+), CD7(+), CD20(+), UCHL-1(+), PAX5(+), Bcl-2(+), MIB-1(+).	CR (3 years)
14 (8)	83/F	Left cervical lymphadenopathy	CHOP × 3	CD2(+), CD3(+), CD5(+), CD8(+), CD10(+), BCL-6(+), CXCL13(+), PD1(+), CD20(dim, +), CD79a(dim, +).	Died (5 months)
15 (9)	59/M	Generalized lymphadenopathy, splenomegaly	CHOPE × 6, R-DHAP × 2, R-GEMOX × 3	CD3(+), CD5(+), CD4(+), TIA1(+), CD20(+), CD79a(+), CD30(rare, +), Ki-67 index:70%.	Died (8 months)
16 (10)	72/F	Eyelid tumor, swelling and erythema of the central lower forehead and glabellar region	Initially suspected lupus; treated with hydroxychloroquine for 6 months, tumor enlarged; COP-E × 6	CD3(+), CD7(+), CD2(+), CD4(+), CD8(+), CD20(+), CD68(+), Ki-67 index:80%–85%.	Died (1 months post-chemo)
17 (11)	68/F	Upper abdominal discomfort	R-CDOP × 5	CD3(+), CD4(+), CD5(+), CD7(+), CD20(+), CD30(+), CD79(+), Bcl-2(+), Bcl-6(+), c-MYC(+), MUM1(+), PAX5(+), Ki-67 index: 70%.	PR(after five cycles)
18 (12)	83/F	Generalized scattered plaques and red nodules	R-CHOP × 3;R-ESHA × 1; steroids + etoposide	CD3(+), CD4(+), CD5(+), CD8(+), CD20(+).	Alive (1 year), with a large ulcerative tumor on the right leg
19 (13)	69/M	Abdominal mass, hypercalcemia	MOPP	CD2(+), CD3(+), CD4(+), CD5(+), CD7(+), CD1a(dim, +), CD20(+)	Died (2 months)
20 (14)	47/M	Cervical lymphadenopathy; history of nodular Hodgkin lymphoma, in CR for 5 years	M-BACOP × 6	CD3(+), CD4(+), CD5(+), CD15(+), CD20(+), CD30(+), CD43(+), CD45RB(+), CD45RO(+), perforin(+).	DFS (7 months)
21 (15)	84/M	Generalized superficial lymphadenopathy	CHOP × 1, COP × 2, radiotherapy	CD2(+), CD3(+), CD4(+), CD5(+), CD20(dim, +), CD25(+), D38(+), CD45(+), CD52(+), Ki-67 index:30%–50%.	Died (6 months)
22 (16)	65/F	Posterior thigh nodule	Radiotherapy, R × 4, radiotherapy, CHOP	CD2(+), CD3(+), CD4(+), CD5(+), CD8(+), CD10(+), CD20(+), CD43(+).	Recurrent Disease (3 years)
23 (17)	38/M	Cervical mass	CHOP × 4	CD20, CD3, CD5, CD7(dim, +), TIA-1(dim, +), MIB 1(15%, +).	Alive (6 months)
24 (18)	65/M	Cervical lymphadenopathy	CHOPE × 1, DICE + Chidamide × 1, hyper-CVAD + Chidamide × 1, R-pGEMOX × 3, Radiation, Pemetrexed + Topotecan × 2	CD20(+), CD3(+), Bcl-2(+).	Died (14 months)
25 (19)	75/M	Left axillary lymphadenopathy	R-CHOP × 6, ICE × 3	CD3(+), CD7(+), CD19(+), CD20(+), CD79a(+).	CR (after progression)
26 (20)	74/F	Uterine cervical tumor for 2 months, gradually enlarging. Generalized lymphadenopathy	CHOP × 6, ESHAP × 4, R × 1	CD2(+), CD3(+), CD4(+), CD5(+), CD7(+), CD8(+), CD10(+), CD20(+), CD56(+), CD79a(+).	Died (12 months)
27 (21)	59/M	Right testicular enlargement	R-CHOP × 6, high-dose methotrexate	CD3(+), CD5(+), CD7(+), granzyme B(+), TIA1(+).	Died (CNS infiltration, 16 months)
28 (22)	79/M	Generalized lymphadenopathy	R-CHOP × 6, GDP	CD3(+), CD4(+), CD8(+), CD20(+), CD45RO(+).	Died
This case	62/F	Posterior tongue lesion	CHOPE × 4	CD3(+), CD2(+), CD5(+), CD43(+) CD20(+), BCL-2(+), CD79α(+), CD10(+), PAX-5(+), CD19(+), Ki-67 index:30%.	CR (5 months)

HSCT, hematopoietic stem cell transplantation; CNS, central nervous system; AWD, alive with disease; PR, partial remission; DFS, disease-free survival; mets, metastases.

Of particular note, a patient in our study achieved a complete response (CR) at the interim assessment following a combined-modality approach of surgery and CHOPE chemotherapy. This stands in contrast to the high overall mortality rate generally observed with this regimen. An in-depth analysis of this patient’s clinical features revealed a constellation of highly favorable low-tumor-burden and low-risk factors, including: (1) Early diagnosis: Localized, Ann Arbor stage I disease; (2) Localized lesion: A primary tongue lesion with a maximum diameter of ≤4 cm; (3) Curative-intent surgery: Achievement of an R0 resection; (4) No residual metabolic activity: Post-operative PET/CT showed a Deauville score of 1; and (5) Low-risk prognosis: An International Prognostic Index (IPI) score of 1 (no B symptoms, normal LDH, and an ECOG performance status of 0). This unique observation gives rise to a hypothesis worthy of investigation: for a select subgroup of CD20+ PTCL-NOS patients who present with localized, resectable disease and a low-risk profile, could a combined-modality therapy of surgery followed by CHOPE chemotherapy represent an effective therapeutic strategy? It must be emphasized that, as this is a single case report with limited follow-up, the current state of complete response can only be considered a preliminary and positive therapeutic reaction, the long-term durability of which remains to be determined. Therefore, the primary value of this report lies not in recommending a specific treatment regimen, but in highlighting the potential clinical heterogeneity of this disease. It suggests that in future clinical practice and research, precise risk stratification for each patient to identify subgroups who may benefit from multi-modality treatment could be more critical than selecting a chemotherapy regimen based solely on pathological subtype.

## Data Availability

The raw data supporting the conclusions of this article will be made available by the authors, without undue reservation.
